# Rehabilitation of Malpositioned Implant in the Anterior Region With Customized Abutment

**DOI:** 10.7759/cureus.50450

**Published:** 2023-12-13

**Authors:** Ankita Pathak, Mithilesh M Dhamande, Seema Sathe, Anjali Bhoyar, Smruti Gujjelwar

**Affiliations:** 1 Prosthodontics, Sharad Pawar Dental College and Hospital, Datta Meghe Institute of Higher Education and Research, Wardha, IND

**Keywords:** anterior aesthetics, customized abutment, prosthodontists, implantology, malpositioned implant

## Abstract

The misplacement of implants represents an unfavorable complication in dental implant prostheses. Numerous instances necessitating restorative intervention, colloquially termed "rescue cases," encompass either novel occurrences or reconstructions of irreversible implant scenarios. A viable solution for addressing these prosthesis-related challenges is using cementable options. This issue is directly linked to improperly placed implants, which dentists find challenging to rectify due to the absence of angle-correcting abutments from manufacturers. In such situations, the preferred treatments involve either Implant Innovations' universal castable long abutment (UCLA) (Palm Beach Gardens, Florida, United States) or a customized abutment fashioned from the titanium alloy impression coping for the Core-Vent implant (Paragon, Calabasas, California, United States). The Department of Prosthodontics received a referral for a patient with an anterior implant that had been misplaced and required rehabilitation. This case report outlines the chosen treatment approach for an implant situated buccally.

## Introduction

The most prevalent reason patients desire improved cosmetic and functional teeth in clinics is tooth replacement. Missing teeth can be replaced with prosthetics that enhance patient satisfaction and facilitate effective chewing while preserving dental health and integrity [1].

Dental implant restorations have emerged as a reliable option in dentistry, offering a predictable treatment approach. However, in some instances, the implant is misplaced, leading to prosthetic challenges. Despite significant advancements, complications are prevalent in implant-supported restorations. These issues may stem from inadequate treatment planning, case selection, insufficient communication among the patient, surgeon, prosthodontist, and laboratory personnel, as well as flawed operator techniques, among other factors. Improperly placed dental implants during surgery represent a commonly encountered clinical complication. Managing prosthetics for cases involving misaligned or malpositioned implants presents a challenge for both prosthodontists and laboratory personnel. Various techniques, such as angulated abutments, castable abutments, and, in severe instances, removable prostheses, have been introduced to address these challenges [2].

The growing research in dental implantology has led to an increased application of dental implants in diverse clinical scenarios. Complications arise with the rising frequency of dental implant placements, particularly in dealing with the challenge of malpositioned dental implants. Rehabilitating prosthetically challenging implants poses a significant challenge for restorative dentists, laboratory technicians, and patients, especially in the critical maxillary smile zone. Prosthetic adjustments, surgical corrections, or a combination of both may be necessary in such cases [3-5].

The rationale of this clinical report is that the success of an implant extends beyond achieving osseointegration; it also hinges on its proper placement and the creation of a harmonious, naturally blending prosthesis. However, situations may arise where the misaligned placement of the implant and limited interarch space for future prostheses become problematic for the prosthodontist, particularly in the aesthetic zone. In these intricate cases, restoration with a tailored treatment plan becomes essential to meet the aesthetic preferences of patients while ensuring the successful restoration of the implant with a fixed prosthesis [6,7].

In such a case, prefabricated, regularly available abutments won't serve the purpose. The customized abutments are designed and cast in such a way that the emergence profile is improved and the loaded prosthesis will conform to the arch form. The selection of abutments is critical to the success of a fixed prosthesis. Customized abutments can be employed for cement- or screw-retained single crowns or bridges. Abutments can alter the force transmission to the surrounding bone by adjusting the implant stress and strain distribution. In constructing an implant-supported prosthesis, parallel abutments are frequently used. However, in some cases, further surgical operations may be necessary to secure an implant in an optimum tridimensional position [8-10]. Hence, this case report describes the prosthetic management of malpositioned implants in the anterior aesthetic zone. 

## Case presentation

**Figure 1:**
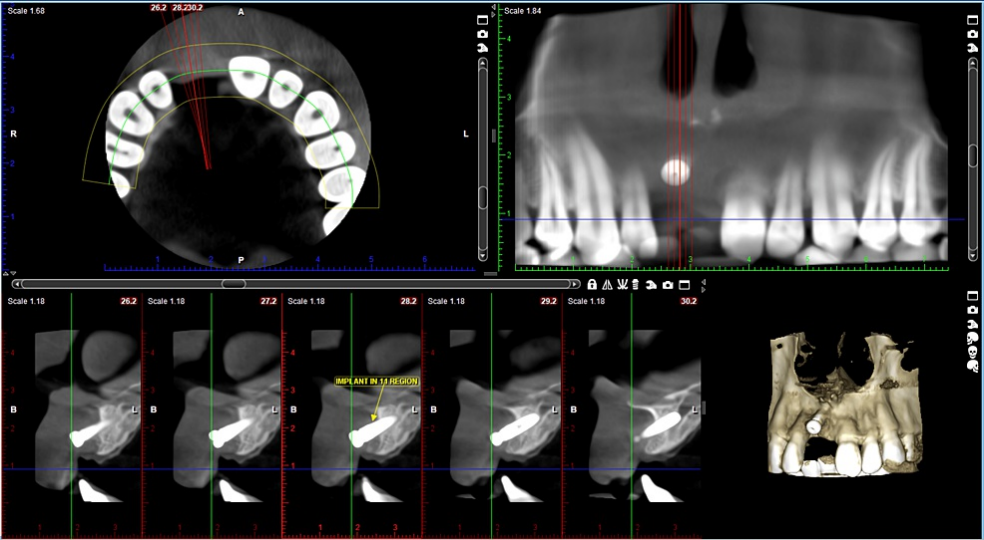
CBCT showing malpositioned implant in 11 region CBCT: cone beam computed tomography Image Credit: Ankita Pathak

**Figure 2:**
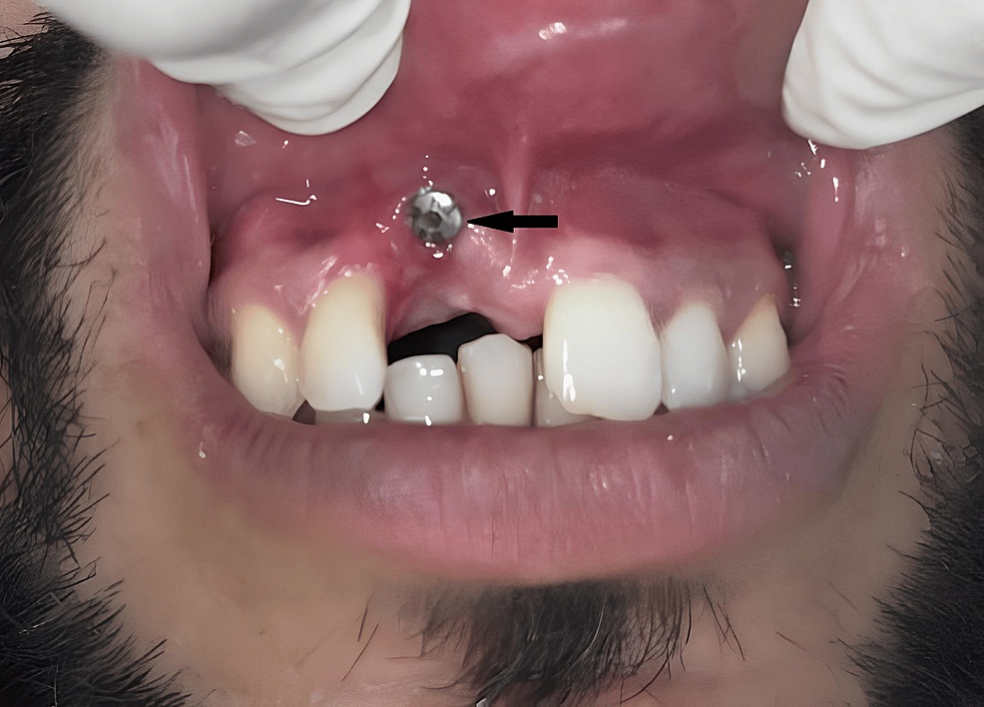
An implant buccally placed in the anterior region Image Credit: Ankita Pathak

A 32-year-old male patient was referred from the Department of Oral Surgery. The patient was asymptomatic two years ago, after which he met with a road traffic accident, resulting in the loss of his permanent right central incisor. Since the patient insisted on a fixed solution, it was planned to place an implant in the position of the right central incisor. Due to significant bone loss, the implant was placed buccally. However, it was observed radiographically and intraorally that the implant was positioned too buccally, outside of the arch, as shown in Figure 1 and Figure 2.

**Figure 3:**
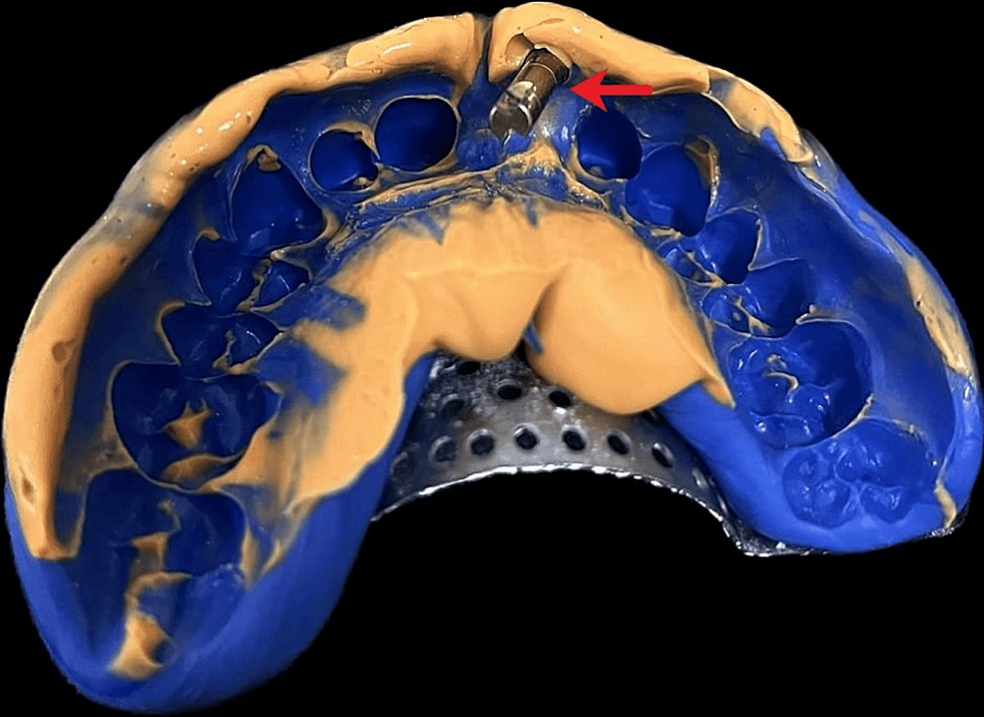
Impression made in 11 region Image Credit: Ankita Pathak

The patient had no significant past medical history. A thorough assessment and evaluation were conducted using diagnostic models and available radiographs. The patient was mainly concerned about aesthetics. Despite the compromised placement of the implant, a decision was made to load the implant with a customized abutment in accordance with the patient's preferences, following the acquisition of informed consent from the patient. Rehabilitating the implant with regularly available abutments proved challenging. To avoid gingival impingement by conventional abutments, the option of a customized abutment was considered. It was decided that the treatment of choice would be to rehabilitate this implant with a customized abutment. An impression coping was attached for a mini implant, and an impression was made using putty and light-body polyvinyl siloxane material. Subsequently, a cast was fabricated after attaching a lab analog, as depicted in Figure 3.

**Figure 4:**
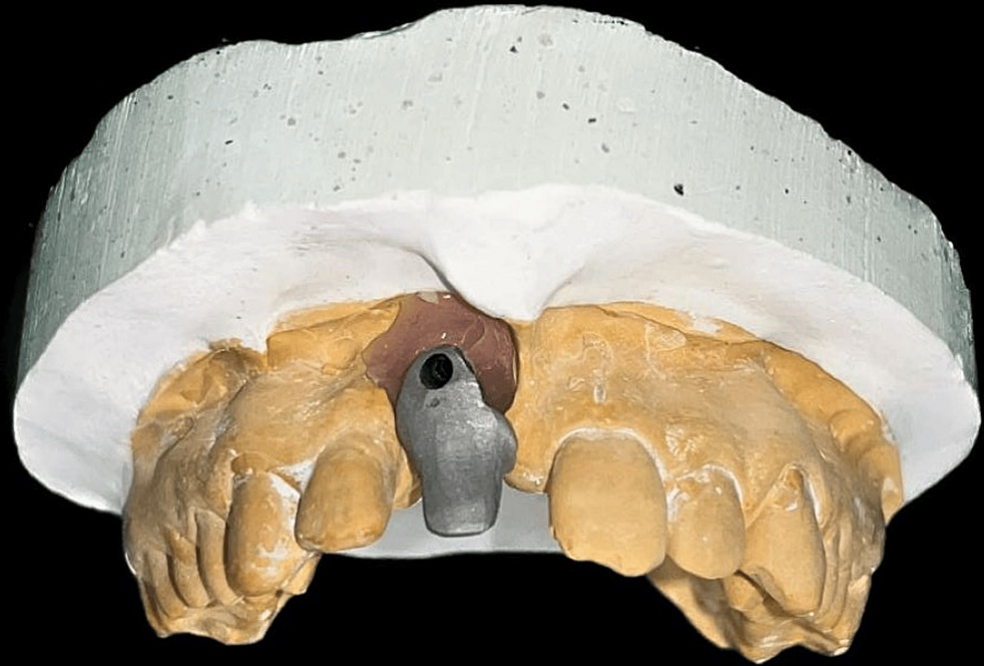
Buccal view of the customized abutment Image Credit: Ankita Pathak

**Figure 5:**
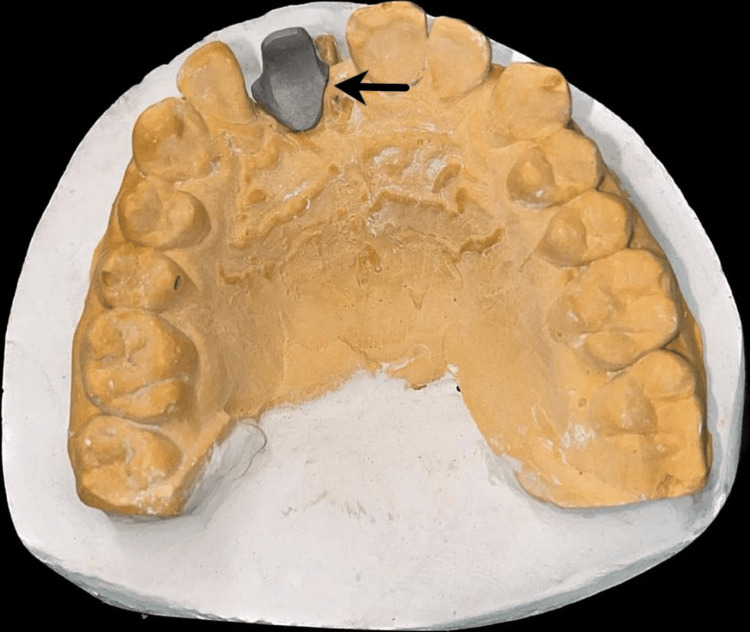
Palatal view of the customized abutment Image Credit: Ankita Pathak

Gingival or aesthetic mask was applied over the impression in 11 region to replicate soft tissue in the cast. The fabrication process of the customized abutments involved creating a wax pattern, which was then cast into metal, as illustrated in Figure 4 and Figure 5.

**Figure 6:**
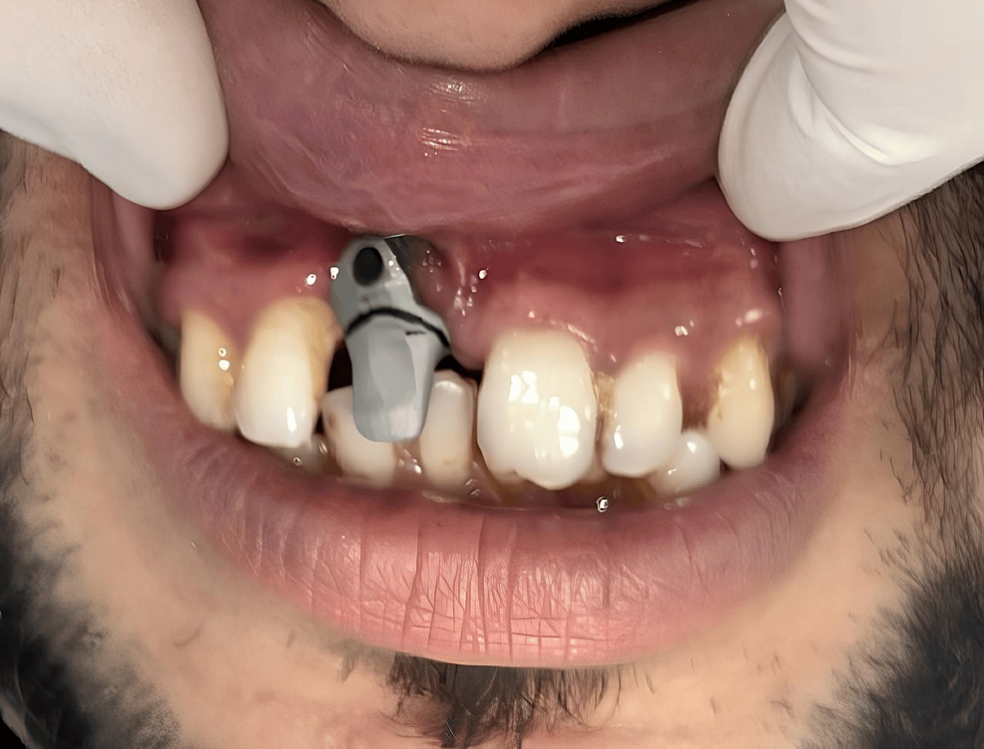
Intraoral metal try-in to show the marking of gingival porcelain Image Credit: Ankita Pathak

For the casted abutment, an intraoral metal try-in was conducted, and the level for the marginal gingiva was marked, as depicted in Figure 6.

**Figure 7:**
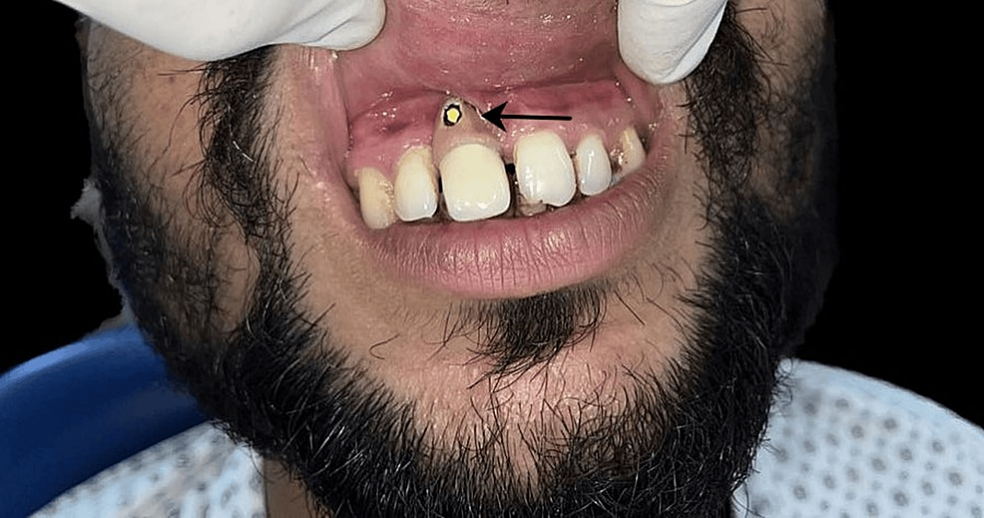
PTFE is placed and packed in the access hole of the abutment PTFE: polytetrafluoroethylene Image Credit: Ankita Pathak

**Figure 8:**
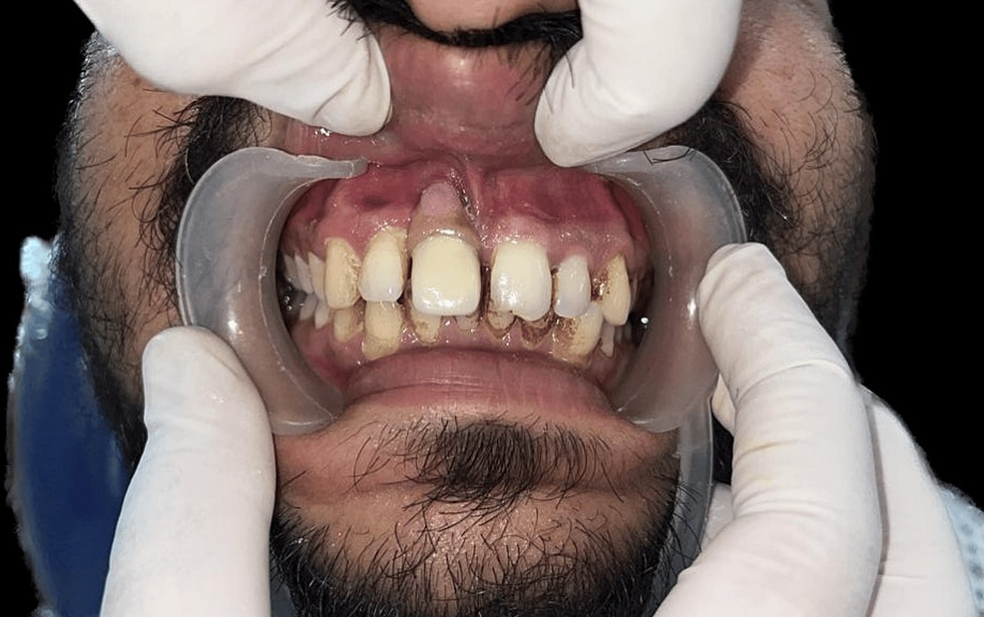
Cementation of prosthesis Image Credit: Ankita Pathak

The shade selection was performed using the Vita classic shade guide (Vita, Yorba Linda, California, United States). A porcelain-fused metal crown was then fabricated over the casted abutment, covering the gingival portion with pink porcelain. Before cementing the crown, all excursive contacts were assessed and removed. The abutment was securely screwed in place, over which the crown was cemented, as depicted in Figure 7 and Figure 8.

## Discussion

The location of the implant is critical for both the aesthetics and functionality of the prosthesis. Comprehensive treatment planning and diagnosis are crucial steps in implant placement. Prosthodontists encounter challenges in aesthetics and function when placing implants in the anterior area. Gingival ceramics can alter the emerging profile of an apical coronally inserted implant [11]. Factors such as surgical stents, radiographic evaluations, soft and hard tissue structures, abutment selection, final prosthesis design, and the patient's systemic status all influence implant placement [12].

In this case, the implant was positioned too buccal in proportion to the remaining teeth. Two alternatives were presented to the patient: either to remove the incorrectly placed implant, followed by a fixed prosthesis or the insertion of a fresh implant in the correct location, or to load the implant with a permanent prosthesis to sequentially mold the gingiva surrounding the implant. The patient declined to have the incorrectly placed implant removed. In cases where the patient's implant is misaligned, prefabricated or custom-made angled abutments can be used to restore function, comfort, and aesthetics [13,14].

Aesthetic failures in dental implant procedures can be attributed to poor prosthesis shade selection and a lack of interdental papillae. Therefore, among the characteristics deemed essential for success in dental implant rehabilitation, the prosthesis constructed over the implant must be satisfactory to both the dentist and the patient. Examples of such considerations include larger crowns, artificial gingiva, angled abutments and/or custom porcelain coatings, and secondary grafts [15]. Misalignment of a maxillary anterior implant makes it challenging to retain and replicate the natural mucogingival architecture that surrounds it [16,17].

The implant body should ideally be perpendicular to the Wilson and Spee curves generated by compressive stresses along the implant's longitudinal axis. Axial parallelism minimizes total stress on the implant system, as well as the biological and mechanical problems associated with it, such as abutment fracture and screw loosening. Anatomical variances, such as bone concavities on the maxillary facial aspect or the lingual surface of the jaw, may necessitate the clinician placing the implant at an angle.

In implant dentistry, implant placement with an angulation greater than 25°, as well as the use of customized abutments, is potentially hazardous. Despite advances in computer-aided design and production techniques, casting remains an accepted method for generating these abutments. When the inter-occlusal space is restricted, implants are placed outside of the arch, and the implant angle exceeds 30°, cast abutments can be employed. These abutments are also economically priced [18,19]. Cast abutments are classified into two types: cast-to and castable. The primary benefit of cast-to abutments is the connection's perfect fit. The connecting region, on the other hand, is cast with the castable type, resulting in lower accuracy [20].

Limitation

Multiple investigations on the effect of abutment rotational flexibility on implants have discovered that abutment movement on the implant may lead to screw loosening [21]. Limitation of the abovementioned case is the faulty angulation and placement of implant. Also, the long-term success is questionable as the angulation abutment and implant junction may lead to untoward forces and ultimately can cause failure of the implant. Binon found that increasing the rotational flexibility of the screw from 2° to 3° reduced the cycle in cyclic loads before screw loosening by 26% [22]. Screw loosening is mostly the result of implant component mismatch, inadequate tightness, putting excessive stresses on the complex, and poor screw design. Other reasons for screw loosening include screw bending stresses, the settling effect, and a decrease in preload [23]. In this report, the implant is in a very unfavorable location, and its long-term success is severely undermined. Yet all efforts have been made to increase the longevity of the implant by removing the excursive contacts. 

## Conclusions

The fabrication of customized castable abutments is considered an option rather than a solution for managing implant-supported prostheses in such cases. Diagnosis and treatment planning plays a vital role in the successful restoration of implants. As the implant was positioned outside of the arch, rehabilitating such an implant is challenging and demands special attention. A customized abutment is the preferred treatment in such cases. The significance of clinical precision is underscored by the buccally placed access hole of the implant, necessitating the application of pink porcelain or gingival porcelain to effectively camouflage the site. This nuanced approach acknowledges the aesthetic considerations crucial for patient satisfaction. In the aforementioned case, malpositioned implants can be rectified through prosthetic rehabilitation using customized castable angled abutments.
